# DSTEELNet: A Real-Time Parallel Dilated CNN with Atrous Spatial Pyramid Pooling for Detecting and Classifying Defects in Surface Steel Strips

**DOI:** 10.3390/s23010544

**Published:** 2023-01-03

**Authors:** Khaled R. Ahmed

**Affiliations:** School of Computing, Southern Illinois University, Carbondale, IL 62901, USA; khaled.ahmed@siu.edu

**Keywords:** computer vision, defect detection, defect classification, parallel processing, convolution neural network

## Abstract

Automatic defects inspection and classification demonstrate significant importance in improving quality in the steel industry. This paper proposed and developed DSTEELNet convolution neural network (CNN) architecture to improve detection accuracy and the required time to detect defects in surface steel strips. DSTEELNet includes three parallel stacks of convolution blocks with atrous spatial pyramid pooling. Each convolution block used a different dilation rate that expands the receptive fields, increases the feature resolutions and covers square regions of input 2D image without any holes or missing edges and without increases in computations. This work illustrates the performance of DSTEELNet with a different number of parallel stacks and a different order of dilation rates. The experimental results indicate significant improvements in accuracy and illustrate that the DSTEELNet achieves of 97% *mAP* in detecting defects in surface steel strips on the augmented dataset GNEU and Severstal datasets and is able to detect defects in a single image in 23ms.

## 1. Introduction

Quality control is the key success aspect of steel industrial production [1,2,3]. Surface defect detection is an essential part of the steel production process and has significant impacts upon the quality of products. Manual defect detection methods are time-consuming and subject to hazards and human errors. Therefore, several traditional automatic surface defect detection methods have been proposed to overcome the limitations of manual inspection. These include eddy current testing, infrared detection, magnetic flux leakage detection, and laser detection. These methods failed to detect all the faults, especially the tiny ones [4]. This motivated researchers [5,6,7,8] to develop computer vision systems that are able to detect and classify defects in ceramic tiles [5], textile fabrics [9,10] and steel industries [7,8,9,11,12]. Structure-based methods extract image structure features such as texture, skeleton and edge, while other methods succeed to extract statistical features, such as mean, difference and variance [13], from the defect surface and then apply machine learning algorithms to train these features to recognize defected surfaces [14,15]. The combination of statistical features and machine learning achieves higher accuracy and robustness than structure-based methods [16]. Using machine learning, such as Support Vector Machine (SVM) classifier to classify different types of surface defects may take approximately 0.239 s to extract features from a single defect image during testing [14]. Therefore, it fails to meet the real-time surface defect detection requirements. However, convolutional networks (CNN) provide automated feature extraction techniques that take raw defect images and predict surface defects in a short time and lessen the requirements to manually extract suitable features [17,18,19]. The deep learning models for surface defects classification are more accurate than traditional image processing-based and machine learning methods. Defects in the surface steel strips have multiple of challenges, such as (1) low contrast due to change of light intensity, (2) defects are similar to background, (3) irregular shape of defects, (4) multiple scales of defects of the same kind, and (5) there are insufficient training samples. These challenges degrade the accuracy of the deep learning model. Therefore, to detect and classify defects of different sizes, other research efforts integrated multi-scale features with image classification CNN networks throughout successive pooling and subsampling layers [20,21,22,23]. The use of multi-scale features reduces resolution until obtaining a global prediction. To recover the lost resolutions different approaches have been designed, such as using repeated up-convolutions, atrous spatial pyramid pooling (ASPP) module and using multiple rescaled versions of the image as input to the network while combining the predictions obtained for these multiple inputs [24,25,26,27]. 

The main objective of this research is to enhance steel strips surface defects detection accuracy and produce a significant prediction model. Therefore, in response to the above challenges, we proposed a CNN, called *DSTEELNet* for detecting and classifying defects in surface steel strips that aggregates different feature maps in parallel without losing resolution or analyzing rescaled images [28]. The proposed module is based on parallel stacks of different dilated convolutions that support exponential expansion of the receptive field without loss of coverage or resolution. The dilated convolution can capture more distinctive features by shifting the receptive field [29], and able to gather multi-scale features. This paper investigates the performance of the proposed *DSTEELNet* with different number of parallel stacks and different dilation rates per stack. In addition, the author employs a specific order of dilated convolutions in *DSTEELNet* to cover square regions of input 2D image without any holes or missing edges. The main contributions of this paper are as follows: (1) We proposed and developed a novel framework called *DSTEELNet* that includes three parallel stacks of dilated convolution blocks with different dilation rates, which significantly enhance the inference speed and the detection accuracy of defects for surface steel strips. They are able to capture, propagate different features in parallel and cover square regions of input 2D image without any holes or missing edges; (2) We evaluated the proposed *DSTEELNet* architecture and the traditional CNN architectures on NEU [3] and Severstal [30] datasets to highlight the effectiveness of *DSTEELNet* in detecting and classifying defects in surface steel strips; (3) We proposed and developed the *DSTEELNet-ASPP* that adopts the atrous spatial pyramid pooling (ASPP) module [27] to enlarge the receptive field and incorporate multi-scale contextual information without sacrificing spatial resolution; and (4) We used a deep convolution generative adversarial network *DC*GAN to extend the size of the NEU dataset and consequently improve the performance of the generated models.

The rest of this paper is organized as follows. Section 2 reviews the related works. Section 3 illustrates the training datasets, augmentation techniques, the proposed DSTEELNet CNN framework, and demonstrates the experiments setup and performance metrics. Section 4 discusses the experimental results. Section 5 concludes this paper and provides the future research direction.

## 2. Related Work

There are several research efforts that have developed machine vision techniques for surface defect detection. They are mainly divided into two categories, namely: the traditional image processing method, and the machine learning methods. The traditional image processing methods, detect and segment defects by using the primitive attributes reflected by local anomalies. They detect various defects by feature extraction techniques that are categorized into four different approaches [31,32,33]: structural method [34,35], threshold method [36,37,38], spectral method [39,40,41], and model-based [42,43] method. In traditional image processing methods, multiple thresholds to detect various defects are needed and are very sensitive to background colors and lighting conditions. These thresholds need to be adjusted to handle different defects. The traditional algorithms require plenty of labor to extract handcrafted features manually [13]. Machine learning-based methods typically include two stages of feature extraction and pattern classification. The first stage analyzes the characteristics of the input image and produces the feature vector describing the defect information. These features include grayscale statistical features [44], local binary patterns (LBP) feature [45], histogram of oriented gradient (HOG) features [46], and gray level co-occurrence matrix (GLCM) [44]. Some research efforts have been developed to speed up the features extraction process in parallel using GPU as our previous research work in [47]. The second stage feeds the feature vector into a classifier model that trained in advance to detect whether the input image has a defect or not [16]. In a complex condition, handcrafted features or shallow learning techniques are not sufficiently discriminative. Therefore, these machine learning-based methods are typically dedicated for a specific scenario, lacking adaptability, and robustness.

Recently, neural network methods have achieved excellent results in many computer vision applications. Convolutional neural networks (CNN) have been used to develop several defect detection methods. Some of the CNN research efforts have been developed to classify the defects in steel images as in [11], authors employed a sequential structured CNN for feature extraction to improve the classification accuracy for defect inspection. They did not consider the effects of noise and the size of the training dataset. Authors in [48] developed a multi-scale pyramidal pooling network for the classification of steel defects. Authors in [49] developed a flexible multi-layered deep feature extraction framework. Both research work succeeded in classifying defects, however they failed to localize the location of the defects. Therefore, researchers convert the surface defect detection task into an object detection problem in computer vision to localize defects as in [50]. In [51] authors developed a cascaded autoencoder (CASAE) that first locates defect and then classifies it. In the first stage, it localized and extracted the features of the defect from the input image. In the second stage, it used compact CNN to accurately classify defects. The authors in [50] developed a defect detection network (DDN) that integrates the baseline *ResNet34, ResNet50* [52] networks and Region proposal network (RPN) for precise defect detection and localization. In addition, they proposed the multilevel-feature fusion network that combined lower and high-level features. In other words, the inspection task classifies regions of defects instead of a whole defect image. The authors claimed that *ResNet34* and *ReNet50* achieved of 74.8%, 82.3% *mAP*, respectively, at 20 FPS (frames per second) [50]. The research work in [53] employed traditional CNN with a sliding window to localize the defect. In [54] authors developed a structural defect detection method based on *Faster R-CNN* [55] that is succeeded to detect five types of surface defects: concrete, cracks, steel corrosion, steel delamination, and bolt corrosion. Recently, authors in [56] reconstructed the network structure of two-stage object detection (Faster R-CNN) for small features of the target, replaced part of the CNN with a deformable convolution network [57] and trained the network with multiscale feature fusion on NEU dataset [3]. This work achieved low mAP of 75.2% and long inference speed. These models able to achieve high defect detection accuracy but low detection efficiency that cannot meet the real-time detection requirements of the steel industry. In addition, researchers in [58] developed single-stage object-detection module named Improved-YOLOv5 that precisely positioning of the defect area, crop the suspected defect areas on the steel surface and then used the Optimized-Inception-ResnetV2 module for defect classification. This works achieved the best performance of 83.3% *mAP* at 24 FPS.

In summary, the limitations of the stated research efforts are that they detect defects through one or multiple close bounding boxes but cannot identify the boundary of the defect precisely in real-time. They have shown acceptable levels of precision, but fail to achieve real-time defect detection requirements in the steel industry. The main aim of this paper is to (1) develop a real-time deep learning framework that accelerates the defect detection speed and improves the detection and classification precision to facilitate quality assurance of surface steel manufacturing; (2) enlarge the training dataset to avoid overfitting. Annotating the data collected from the manufacturing lines is a time-consuming task. To address this issue, there has been recent interest in the research community to mitigate it. The next section illustrates the (1) data augmentation techniques used to enlarge the NEU dataset and (2) proposed deep CNN architecture.

## 3. Materials and Methods

This section illustrates the training datasets, augmentation techniques, and the proposed *DSTEELNet CNN* framework to classify and detect surface defects in real-time. Finally, it demonstrates the experiments setup and performance metrics.

### 3.1. Datasets 

**Figure 1:**
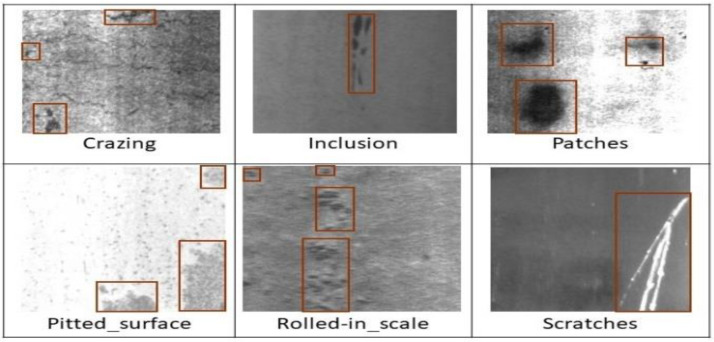
Six types of surface steel strips defect.

For training and experiments, we used two steel surface NEU [3] and Severstal [30] datasets. This section introduces the NEU dataset and the expansion techniques in detail to facilitate the training of the proposed model. In our experiment, we used NEU dataset [3]. Originally, the NEU dataset has 1800 grayscale steel images and includes six types of defects as shown in Figure 1. The defect types are crazing, inclusion, patches, pitted surface, scratches, and rolled-in scale, 300 samples for each type. To annotate the dataset, each defect that appears in the defected images is marked by a bounding red box (groundtruth box) as shown in Figure 1. Approximately 5000 groundtruth boxes have been created. These bounding boxes were used only to localize defects. They were not used to represent either defect’s borders or describe their shape. In addition, we trained the proposed model using Severstal dataset that includes 12,568 training steel plate images, 71,884 pixel-wise annotation masks among four different types of steel defects. The defect types are defect 1 (Pitted surface), defects 2 (Inclusion), defects 3 (Scratches), and defects 4 (Patches) as classified in NEU. 

#### 3.1.1. NEU Dataset Augmentation 

The NEU dataset includes a small quantity of training samples and image-level annotation labels that are not adequate to provide sufficient information for industry applications. To expand the dataset with new samples, a naive solution to oversampling with data augmentation would be a simple random oversampling with small geometric transformations such as 8° rotation, shifting image horizontally or vertically, etc. There are other simple image manipulations such as mixing images, color augmentations, kernel filters, and random erasing can also be extended to oversample data as geometric augmentations. This can be useful for ease of implementation and quick experimentation with different class ratios. In this paper, we used data augmentation to manually increase the size of the NEU dataset by artificially creating different versions of the images from the original training dataset. Table 1 shows the images augmentation setting parameters used to generate augmented images such as flip mode, zoom range, width shift, etc. For example, width shift was used to shift the pixels horizontally either to the left or to the right randomly and generate transformed images. The generated images have been combined with the original NEU dataset. However, oversampling with basic image transformations may cause overfitting on the minority class which is being oversampled. 

The biases present in the minority class are more prevalent post-sampling with these techniques. Therefore, this paper also used neural augmentation networks such as Generative Adversarial Network (GAN) [59] to generate a new dataset called *GNEU*. The GAN can generate synthetic defect images that are nearly identical to their ground-truth original ones. Similar to [60], we developed a deep convolution GAN named *DCGAN* that includes two CNNs: generator *G* (reversed CNN) and discriminator *D*. Generator *G* takes random input and generates an image as output from up-sampling the input with transposed convolutions. However, *D* takes the generated images and original images and tries to predict whether a given generated image is (fake) or original (real). The GAN network performs min-max two players game with value function *V*(*D, G*) [59]:

(1) $$min_{G}\;\max_{D}\;V\left(D,G\right),$$

(2) $$\;V\left(D,G\right)=E_{\omega \sim S_{data}\left(\omega \right)}\left[loG\;D\left(\omega \right)\right]+E_{\tau \sim S_{\tau }\left(\omega \tau \right)}\left[loG\left(1-D\left(G\left(\tau \right)\right)\right)\right]\;\;\;$$

where *D*(*ω*) is the probability of ω is a real image, *S_data_* is the distribution of the original data, τ is random noise used by the generator *G* to generate image *G*(*τ*) and *S_τ_* is the distribution of the noise. During training, the aim of the discriminator *D* is to maximize the probability *D*(*ω*) assigned to fake and real images. Since it is a binary classification problem, this model is fit seeking to minimize the average binary cross entropy. Minimax Gan loss is defined as minimax simultaneous optimization of the disseminator and generator models as shown in Equation (1). The discriminator pursues to maximize the average of the log probability for real images and the *LoG* of the inverted probabilities of fake images. In other word, it maximizes the *LoG D*(*ω*) + *LoG*(1−*D*(*G*(*τ*))). The generator pursues to minimize the LoG of the inverse probability predicted by the discriminator for fake images. In other word, it minimizes the *LoG*(1−*D*(*G*(*τ*))). 

##### GAN Architecture

In this paper, we used the similar GAN architecture developed in [60] as follows. Authors in [60] designed a generator *G* that includes first a dense layer with a *ReLU* activation function followed by batch normalization to stabilize GAN as in [59]. To prepare the number of nodes and reshaped into 3D volume, they added another dense layer with the *ReLU* activation function followed by batch normalization. Then, they added a Reshape layer to generate 3D volume from the input shape. To increase the spatial resolution during training they added a transposed convolution (Conv2DTranspose) with stride 2, 32 filters, each of which is *5 × 5*, *ReLU* activation function and followed by batch normalization and dropout of size 0.3 to avoid overfitting. Finally, they added five up-sample and transposed convolutions (Conv2DTranspose), each of which uses stride 2 and *tanh* activation function. These convolutions increased the spatial dimension resolution from *14 × 14* to *224 × 224,* which is the exact of the input images. Afterward, they developed the discriminator *D* as follows. It includes two convolution layers (Conv2D) with stride *2*, *32* filters, each of which is *5 × 5* and *Leaky ReLU* activation function to stabilize training. As well, they added flatten and dense layers with *sigmod* activation function to capture the probability of whether the image is synthetic or real. 

##### Generating GNEU

**Figure 2:**
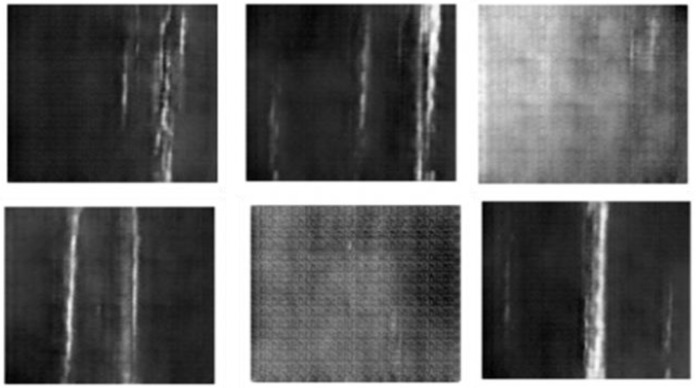
Examples of NEU Synthetic images generated by DCGAN.

We trained the GAN to generate the synthetic images as follows. A noise vector randomly generated using Gaussian distribution and passed to *G* to generate an actual image. Then, authentic images from the training dataset (*NEU*) and the generated synthetic images were mixed. Subsequently, discriminator *D* trained using the mixed dataset with aiming to correctly label each image either fake or real. Again, a random noise generated and labeled each noise vector as real image. Finally, GAN trained using these noise vectors and real image labels even if they are not actual real images. In summary, at each iteration of the GAN algorithm, firstly it generates random images and then trains the discriminator to distinguish fake and real images, secondly it tries to fool the discriminator by generating more synthetic images, finally it updates the weights of the generator based of the received feedback from the discriminator which enable us to generate more authentic images. We stop training GAN after 600 iterations, where the mean of discriminator loss and adversarial loss converge to 0.031 and 1.617, respectively. We mixed the synthetic images with the original NEU images to generate the *GNEU* dataset. Figure 2 shows examples of the results of the generated images from the NEU dataset.

This paper feeds approximately 1800 images of the NEU dataset to the *DCGAN* framework, which generates 540 synthetic images added to the original NEU dataset and creates a new dataset called *GNEU*. We divide *GNEU* dataset into training, validation and testing sets. The training set includes 1260 real and synthetic images, the validation set includes 540 real and synthetic images. The test set includes 540 real images. 

#### 3.1.2. Severstal Dataset 

**Figure 3:**
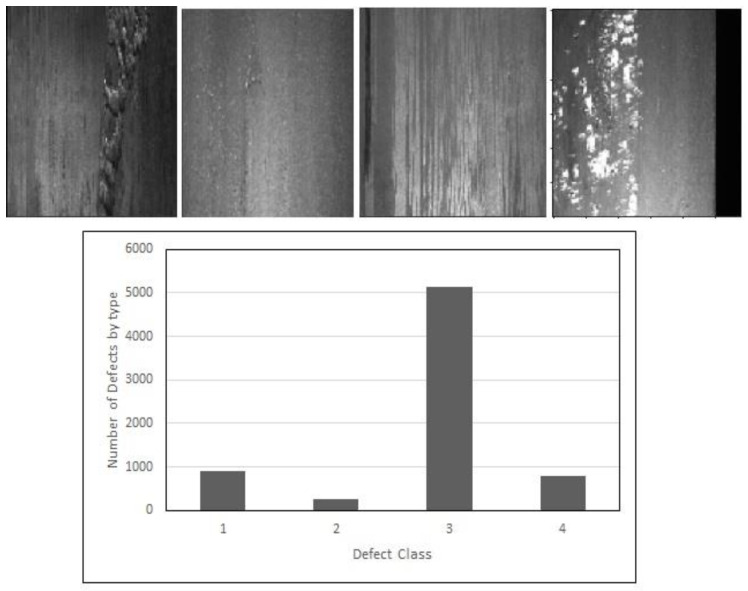
Severstal types of defects.

The Severstal dataset [30] includes approximately 12,568 steel plate training images and 71,884 pixel-wise annotation masks among four different types of steel defects. Figure 3 shows the types of steel defects and the frequency of occurrence of each defect class in the training images. Each steel plate, high resolution image is 256 × 1600 pixels. The training data has 5902 images without defect and 6666 images has defects. Furthermore, the number of images with one label is 6293, with two labels is 425 and 2 images with three labels. Images captured by using high frequency cameras mounted on the production line. The shape of each annotation mask is also 256 ×1600 pixels. Severstal dataset includes four types of surface defects. To annotate defects with small mask file size, the dataset uses run-length encoding (RLE) on the pixel values. The RLE represents the pairs of values that have a start position and a run length. For example, ‘10 5’ means starting at pixel 10 and running a total of 5 pixels (10,11,12,13,14) where the pixels are numbered from top to bottom, then left to right: 1 is pixel (1,1), 2 is pixel (2,1), etc. The evaluation metric required by Severstal is the mean *Dice coefficient* as shown in equation 3 that is used to compare the pixel-wise agreement between a predicted segmentation and its corresponding ground truth.

(3) $$Dice=2\frac{\left|A\cap^{\;}B\right|}{\left|A\right|+\left|B\right|}$$

where *A* is the ground truth and *B* is the predicted set of pixels. |*A*| is the total number of pixels in *A*, the ground truth set of pixels. |*B*| is the total number of pixels in *B*, the predicted set of pixels. *|A∩B|* is the total counts of pixels in both *A* and *B*. When both *A* and *B* are empty then the Dice coefficient equals 1. Since Severstal dataset provides adequate number of images in this paper we did not use any augmentation technique to oversample the dataset. 

### 3.2. Proposed DSTEELNet Architecture

This section describes the proposed *DSTEELNet CNN* framework to detect and classify defects in surface steel strips. The proposed *DSTEELNet* aims to generate high quality training results through achieving fine details of the input 2D images by increasing feature resolutions. Expanding the receptive field ${ℛ}_{ℱ}$ increases the feature resolution, whilst ${ℛ}_{ℱ}$ is the portion of the input image where the filter extracts feature and defined by the filter size of the layer in the CNN [61,62]. To expand the ${ℛ}_{ℱ}$, this paper used dilated convolution [29] with a dilation rate larger than 1, where, the dilation rate is the spacing between each pixel in the convolution filter. Adding the dilation rate to the conv2D kernel decreases the computational costs and expands ${ℛ}_{ℱ}$. Equation (4) shows the form to calculate the receptive field ${ℛ}_{ℱ}$ where *k* is the size of the kernel and *d* is the dilation rate.

(4) $${ℛ}_{ℱ}=d\;\left(k-1\right)+1$$

**Figure 4:**
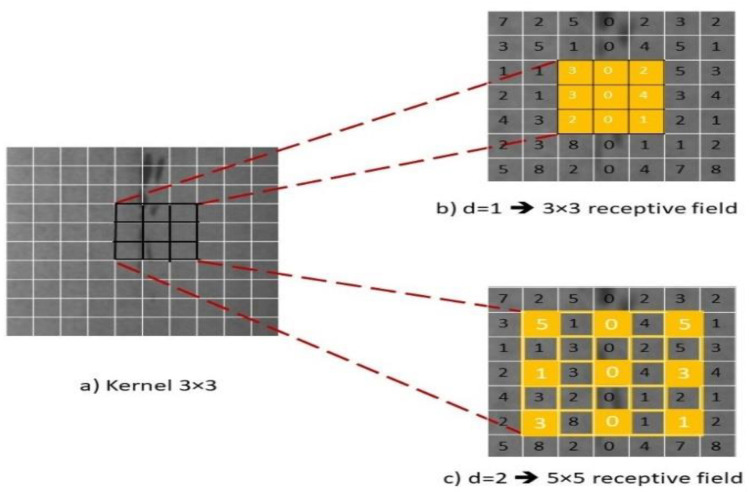
Dilated Convolution in *DSTEELNet*.

For example, using dilation rate of 1 and 3 × 3 kernel generates receptive field with size 3 × 3 which is equivalent to the standard convolution as shown in Figure 4b. The size of the output can be calculated using Equation (5) as follows:

(5) $$\sigma =\frac{g+2p-{ℛ}_{ℱ}}{s}+1$$

where *g × g* input with a dilation factor, padding and stride of *d*, *p* and *s*, respectively. If dilation rate of 2 is used, then each input skips a pixel. Figure 4c. shows 3 × 3 kernel with dilation rate of 2 has the same field of view as 5 × 5 kernel with a gap of *d−1* between. For example, only 9 pixels out of 25 will be only computed around a pixel *x* when *d = 2*, and *k = 3*. As a result, the receptive field ${ℛ}_{ℱ}\;$increased and enabled the filter to capture sparse and large contextual information [63].

The use of systematic dilation expands receptive field ${ℛ}_{ℱ}\;$exponentially without loss of coverage. In other words, the receptive field ${ℛ}_{ℱ}\;$grows exponentially while the number of parameters grows linearly. However, employing a series of dilated convolutional layers with same dilation rate introduced gridding effect problem in which the computations of a pixel in bottom layer are based on sparse/ non-local information. To overcome the gridding effect, the authors in [64] proposed hybrid dilated convolution (HDC) that makes the final size of the ${ℛ}_{ℱ}\;$of a series of convolutional operations fully covers a square region without any holes or missing edges. The HDC developed CNN that includes groups of dilated convolutional layers. Each group has a series of dilated convolutional layers with different dilation rates 1,2,3, respectively. The authors noted that using dilation rate having a common factor relationship (e.g., 2, 4, 8, etc.) in same group of layers may raise the gridding problem. This is contrary to atrous spatial pyramid pooling (ASPP) module [27] where dilation rates have common factors relationships.

**Figure 5:**
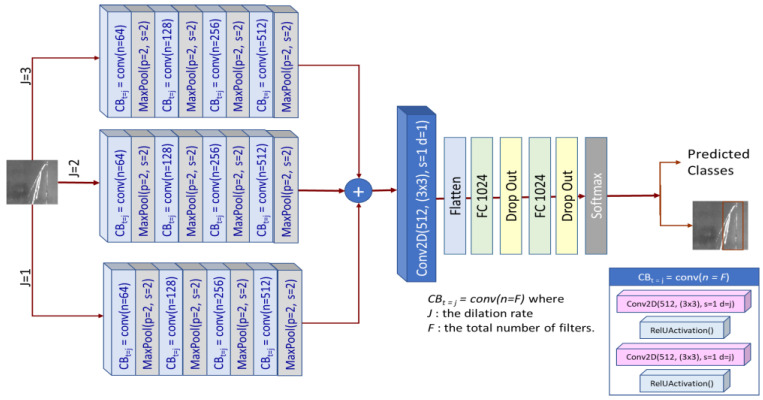
*DSTEELNet* architecture.

In this paper, we developed *DSTEELNet* that includes parallel stacks of dilated convolution with different dilation rates, activation and Max-Pooling layers as shown in Figure 5. At the feature level, we added parallel layers and then performed convolution with activation on the resulting feature maps. We added flatten layer to unstack all the tensor values into a 1-D tensor. The flattened features are used as inputs to two dense layers (Multi-layer perception). To reduce/avoid overfitting, we applied dropout. For classification task, we added dense layer with softmax activation function. Finally, the architecture generates a class activation map. Figure 5 shows the proposed *DSTEELNet* architecture. It includes four dilated convolution blocks in three parallel stacks. Assume each stack includes *m* convolution blocks *CB^(i)^* where i∈{1,2, …m} and the corresponding output of each *CB^(i)^* is denoted by β_i_. The input features and output features are denoted as *f_in_* and *f_out_*, respectively, and *f_out_* can be obtained as follows:

(6) $$f_{out}=f_{in}+\sum_{i=1}^{m}\beta_{i}$$

(7) $$\beta_{i}=\{\begin{array}{cc} CB^{\left(i\right)}\left(f_{in}\right) & i=1\\ CB^{\left(i\right)}\left(f_{in}+\sum_{k=1}^{i-1}\beta_{i-1}\right) & 1<i\leq m \end{array}$$

each convolution block *CB_t=j_ = conv*(*n = F*) followed by Max-pooled block to reduce the feature size and the computational complexity for the next layer. For efficient pooling, we used pool_size = *(2,2)* and strides = *(2,2)* [65]. Each convolution block *CB_t=j_ = conv(n = F)* includes two Conv2D layers followed with *ReLU* activation function where *F* is total number of filters and *j* is the dilation rate. We have used 3 × 3 filters in all convolution blocks. The total number of filters in the first convolution block is *64*, and the rest are *128*, *256*, *512* in order. The three parallel stacks (branches) are similar except they have different dilation rates *j* = *1, 2* and *3*, respectively as shown in Figure 5. We used different dilation rates that have no common factor.

**Figure 6:**
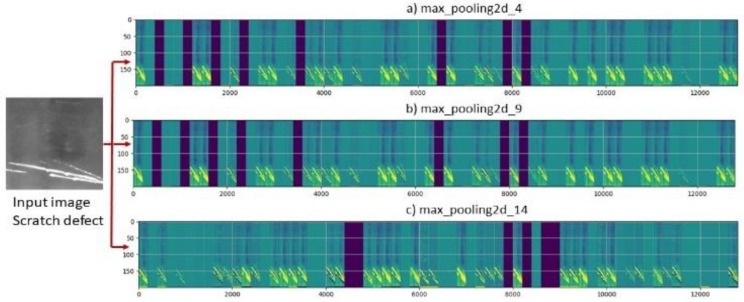
Feature map for three parallel stacks ended with Max_polling layer.

Each parallel branch/stack generates features from images at different CNN layers and then produces different context information as shown in Figure 6. We captured features from the input 2D image using different dilation rates that increases the receptive fields. Figure 6 visualizes *64* output features of three parallel convolutional stacks in Figure 5 with dilation rate *1*, *2* and *3* at layers *max_pooling2d_4*, *max_polling2d_9* and *max_polling2d_14*, respectively. Figure 6a–c shows the features of the input image of size *200×200* in a *200 × (200 × 64*) matrix. The use of parallel stacks with different (i.e., no common factor) dilation rates succeed to cover a square region in the input 2D image without any holes or missing edges. Then, we concatenated the generated features from these parallel branches and handed the resulted features to the next convolution layer to produce the final low-level features. This convolution layer has *512* filters with a filter size *3 × 3*, dilation rate *1*, stride of *1* and followed by *ReLU* activation function. To convert the square feature map into one dimensional feature vector, flatten layer has been added. Two perception (fully connected) layers with size *1024* were used to feed the results of the flatten layer through dense layer that will perform classification. The last dense layer uses *softmax* activation function to determine class scores. To avoid/reduce overfitting during training, a dropout layer has been added to discard some weights produced from two fully connected layers. In this paper, we used dropout of size 0.3.

**Figure 7:**
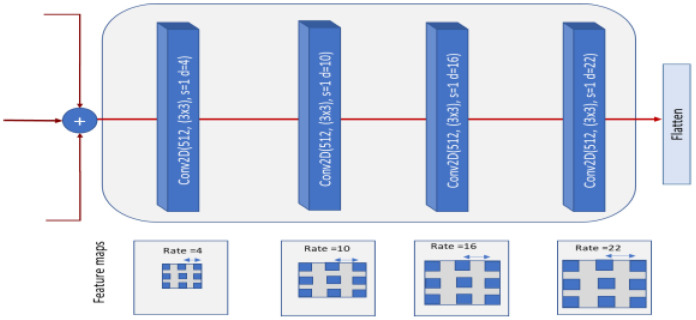
Atrous spatial pyramid pooling module (*ASPP*) replaced the Conv2D layer after concatenating the features in Figure 5. It includes four Conv2D with different dilation rates 4, 10, 16, 22, respectively, and associated feature maps.

For better multi-scale learning and to improve the *DSTEELNet* architecture, we proposed an updated architecture called (*DSTEELNet-ASPP)*. It replaced the Conv2D layer after concatenating the features from the parallel stacks in *DSTEELNet* in Figure 5 by an atrous spatial pyramid pooling (*ASPP*) module [27]. This module includes four Conv2D layers with different dilation rates *4*, *10*, *16*, *22*, respectively to capture defects of distinct size as shown in Figure 7. Then, we concatenated the generated features from these Conv2D layers and handed the resulted features to the flatten layer in Figure 5 to unstack all the tensor values into a 1-D tensor. *DSTEELNet-ASPP* enlarges the receptive field and incorporates multi-scale contextual information without sacrificing spatial resolution. This contributes to improving the overall performance of the *DSTEELNet* architecture.

### 3.3. Experiments

The performance of the *DSTEELNet* is evaluated on the *NEU*, generated dataset (*GNEU*) and Severstal dataset. We demonstrate that *DSTEELNet* achieves a reasonable design and significant results. Therefore, we compare the proposed *DSTEELNet* with state-of-the-art deep leaning detection and classification techniques such as Yolov5, *VGG16*, *ResnNt50*, and *MobileNet*. 

#### 3.3.1. Experiment Metrics

For the performance evaluation, this paper uses the following performance metrics:

(8) $$Recall=\frac{T_{P}}{\left(T_{P}+F_{N}\right)}$$

(9) $$Precision=\frac{T_{P}}{\left(T_{P}+F_{P}\right)}$$

(10) $$AP=\frac{Recall+Precision}{2}$$

(11) $$\;\;\;F1=\frac{2T_{P}}{\left(2T_{P}+F_{N}+F_{P}\right)}$$

(12) $$mAP=\frac{1}{N}\sum_{i=1}^{N}AP_{i}$$

where, *N* is the number of classes, *T_P_* is the number of true Positives, *F_N_* is the number of false Negative, and *F_P_* is the number of false Positive. True positive *T_P_* refers to a defective steel image identified as defective. False positive is referred to defect-free steel image identified as defective. False negative is referred to defective steel image identifies as defect-free. Average Precision *AP* is calculated as the sum of recall and precision divided by two as seen in Equation (10). The F1 score is measured to seek a balance between Recall and Precision. In addition, the mean average precision (*mAP*) is calculated as the average of AP of each class that is used to evaluate the overall performance. 

#### 3.3.2. Experiment Setup

The experiment platform in this work is Intel(R) Core™ i7-9700L with a clock rate of 3.6 GHz, working with 16 GB DDR4 RAM and a graphics card that is NVIDIA GeForce RTX 2080 SUPER. All experiments in this project were conducted in Microsoft Windows 10 Enterprise 64-bit operating system, using Keras 2.2.4 with TensorFlow 1.14.0 backend. We trained the *DSTEELNet*, *DSTEELNet-ASPP*, *VGG16* [66], *VGG19*, *ResNet50* [52], *MobileNet* [67] and *Yolov5* [68] and modified *Yolov5-SE* [69] for approximately 150 epochs on both NEU and *GNEU* training and validation datasets with batch size of 32 and image input size 200 × 200. Similarly, we trained *DSTEELNet*, *VGG16*, *VGG19*, *ResNet50*, and *MobileNet* on *Severstal* dataset where, the image input size is 120 × 120. We applied the Adam optimizer [70] with learning rate 1 × 10^−4^. In addition, we applied the categorical cross entropy loss function in the training. The loss is measured between the probability of the class predicted from *softmax* activation function and the true probability of the category. We did not use any pretrained weights such ImageNet because ImageNet has no steel surface images. We used Equations (8)–(12) to calculate the *AP* per class and the *mAP* for the tested models. 

## 4. Results and Discussion

This section illustrates gradually the results of the proposed CNN architecture to detect defects in surface steel strips. Table 2 demonstrates the weighted average results. It illustrates that *DSTEELNet* performs the highest precision, recall and F1 scores when trained on both NEU and *GNEU* datasets as shown in bold values in Table 2. Additionally, it shows that the use of *DCGN* improved the precision, recall and F-Score of the *DSTEELNet* model by approximately 1%, 1.3% and 1.4%, respectively. Moreover, it shows that *DSTEELNet* outperforms recent CNNs for detecting single defect such as Yolov5 and modified Yolov5-SE [69] by 13.5% and 8.8%, respectively. The Yolov5-SE employs attention mechanism through adding squeeze-and-excitation (SE) block between CSP2_1 and CBL layers to dynamically adjust the characteristics of each channel according to the input. In addition, *DSTEELNet* outperforms the traditional CNNs such as *Vgg16*, *Vgg19*, *ResNet50,* and *MobileNet*.

**Table 2:** Weighted average results on NEU and GNEU datasets.

Model	Precision		Recall		F1-score	
	*NEU*	*GNEU*	*NEU*	*GNEU*	*NEU*	*GNEU*
DSTEELNet	**0.961**	**0.97**	**0.957**	**0.97**	**0.956**	**0.97**
Vgg16	0.91	0.92	0.882	0.89	0.884	0.89
Vg19	0.912	0.92	0.891	0.90	0.894	0.90
ResNet50	0.943	0.95	0.921	0.93	0.92	0.93
MobileNet	0.93	0.94	0.92	0.93	0.92	0.93
Yolov5	*0.821*	*0.835*	*0.836*	*0.84*	*0.836*	*0.84*
Yolov5-SE [69]	*0.879*	*0.882*	*0.887*	*0.89*	*0.886*	*0.89*

Table 3 and Table 4 show the class-wise classification performance metrics listed in Equations (8)–(12). It illustrates the comparison between *DSTEELNet* and the state-of-the-art CNN architectures. Table 3 shows that almost all models tend to enhance the classification of most categories (such as crazing, patches, rolled-in_scale and scratches). The state-of-the-arts models show poor performance to detect defects such as inclusion and pitted_surface due to some similarities in their defect’s structures. However, the *DSTEELNet* succeeded in detecting all the class categories with high accuracy. Table 3 shows that *DSTEELNet* achieves 97.2% *mAP* which outperforms the other models, e.g., VGG16 (91.2%, 6% higher *mAP*), VGG19 (90.0%, 7.2% higher *mAP*), ResNet50 (93%, 4.2% higher *mAP*) and MobileNet (94%, 3.2% higher *mAP*). 

**Figure 8:**
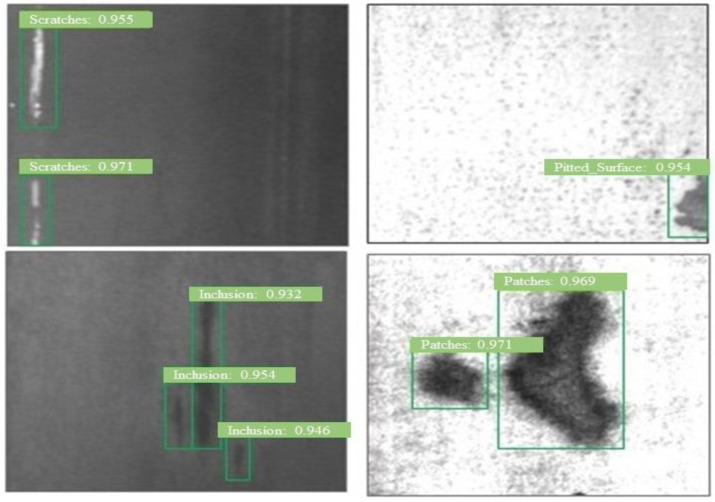
Examples of detection results using *DSTEENet* on GNEU dataset, green box indicating defect location with confidence score.

In addition, Table 3 shows that *DSTEELNet* delivers consistent results for the precision, recall and F1 for crazing, patches, pitted_surface, rolled-in_scale and scratches defects. The *DSTEELNet* succeeds in detecting inclusion defect with highest F1 score (0.91) followed by MobileNet (0.82), ResNet50 (0.79), VGG19 (0.69) and VGG16 (0.68), respectively, in order. Similarly, the *DSTEENet* succeeds in detecting pitted_surface defect with highest F1 score (0.92) followed by MobileNet (0.84), ResNet50 (0.84), VGG16 (0.79) and VGG19 (0.76), respectively, in order. The examples of *DSTEELNet* detection results are shown in Figure 8. It shows that *DSTEELNet* succeeds in detecting defects with significant confidence scores. 

**Figure 9:**
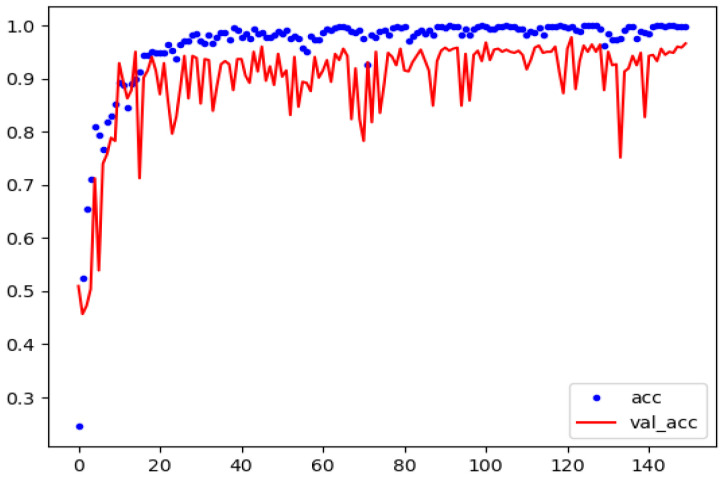
Training and validation accuracy of DSTEELNet on GNEU.

**Figure 10:**
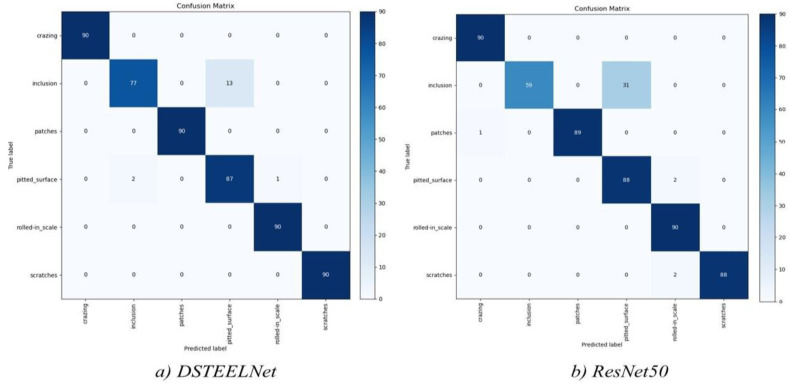
Confusion matrices for *DSTEELNet*, and ResNet50 on test GNEU dataset.

Table 4 depicts a comparative results of single defect classification accuracy with Yolov5 and Yolov5-SE. The low accuracies achieved by Yolov5 and Yolov5-SE to detect small rolled-in-scale defects are badly lowers the average accuracy value. Therefore, *DSTEELNet* outperforms Yolov5 and Yolov5-SE in classifying the six defect types. Figure 9 shows the training and validation accuracy for *DSTEELNet*. It shows that both training and validation accuracy started to improve from epoch 25 and then converged to the highest accuracy values. Figure 10 shows the confusion matrices for *DSTEELNe*t and *ResNet50* evaluated models where the test dataset includes 90 images of each surface defect class. 

Figure 10a shows that *DSTEELNet* detects all the steel surface defects perfectly except the inclusion defects. It misclassified 13 inclusion defects out of 90 as pitted_surface. 

Furthermore, as shown in Figure 10b ResNet50 misclassified 31 inclusion defects out of 90 as pitted_surface. In summary, *DSTEELNet* fails to detect 2.9% of defects in 540 images however, ResNet50, MobileNet, VGG19, and VGG16 fail to detect defects in 6.6%, 7.4%, 10% and 11% of 540 images, respectively. Similarly, to demonstrate that *DSTEELNet* achieves a reasonable design and remarkable results on Severstal dataset, we compare the proposed *DSTEELNet* with *VGG16*, *VGG19*, *ResnNt50*, and MobileNet. Table 5 shows that *DSTEELNet* produces 96% *mAP* which outperforms the other models, e.g., VGG16 (91.2%, 7% higher *mAP*), VGG19 (91.0%, 7% higher *mAP*), ResNet50 (93%, 5% higher *mAP*) and MobileNet (94%, 4% higher *mAP*). 

Table 5 demonstrates the weighted average results on Severstal dataset. It illustrates that for steel surface defect detection *DSTEELNet* performs the highest precision, accuracy and F1 scores as shown in bold values in Table 5.

### 4.1. Dilation Rates Experiments 

**Figure 11:**
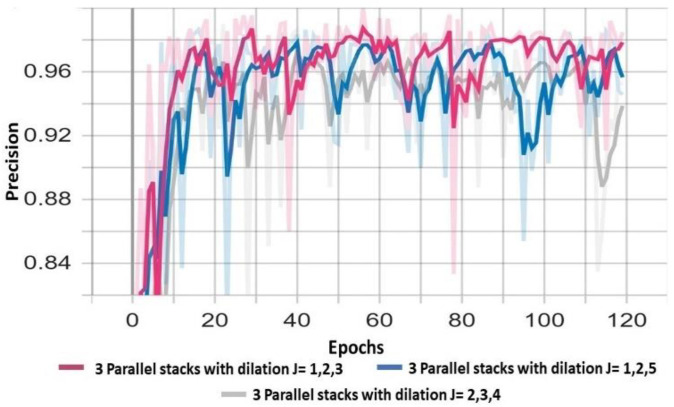
Comparative results of different three parallel stacks with different dilation rates.

The proposed *DSTEELNet* architecture includes four dilated convolution blocks *CB_t=j_* in three parallel stacks. Each stack has a different dilated rate *j* = *1*,*2*,*3*. In this section we examined different *DSTEELNet* architectures through variant dilation rate per stack and number of parallel stacks. We trained the *DSTEELNet* with (1) one stack includes groups of Conv2D layers having different order of dilation rates and (2) three parallel stacks with different dilation rates per stack. Table 6 depicts the weighted average results of different *DSTEELNet* architectures. In Table 6, the use of one stack of Conv2D layers with dilation rates *1*,*1*,*2*,*2*,*3* achieved better results than one stack with dilation rates *1*,*2*,*3*,*4*,*5*. Table 6 and Figure 11 show that using three parallel stacks with dilation rates *1*,*2*,*3* achieved the highest F1-score and precision, respectively. Table 6 shows that the *DSTEELNet-ASSP* improved the precision, recall and F1-score by 2%, 2.2% and 2.1%, respectively, since it enlarges the receptive field and incorporates multi-scale contextual information without sacrificing spatial resolution.

### 4.2. Computational Time 

Table 7 shows the average inference time to detect defects in single image by the proposed technique *DSTEELNet*, and other deep learning and traditional techniques. It reveals that the traditional methods generally are not able to meet the steel industry requirements in real-time. In addition, Table 7 shows that the proposed *DSTEELNet* is the fastest one to detect defects and can meet the real-time requirements. *DSTEELNet* speeds the defect detection time of the traditional techniques by approximately 20 times and outperforms the deep learning techniques. The accuracy of the MobileNet and Resnet50 are higher than *VGG16* and *VGG19*, but they take a longer time to detect defects.

In summary, the *DSTEELNet* achieves the highest accuracy and shortest detection time due to the reduction of its computation complexity. It also outperforms the recent technique called end-to-end defect detection (*EDDN*) [71] that added to Vgg16 extra architectures including multi-scale feature maps and predictors for detection. The authors reported that *EDDN* achieved 0.724 *mAP* and can detect defects in a single image in 27ms. The *DSTEELNet* outperforms EDDN and can detect defects in single image with 0.972 *mAP* at *23*ms. In addition, Yolov5-SE [66] succeeded in detecting defects in a single image with 0.88 *mAP* at 24ms. The *DSTEELNet* succeeds in detecting and classifying defects at 23ms with a higher precision than Yolov5-SE as shown in Table 2 and Table 7. 

## 5. Conclusions

This paper designed and developed a CNN architecture that is suitable for real-time surface steel strips defect detection task. It proposed a *DSTEELNet* that employs sparse receptive fields and parallel convolution stacks to generate more robust and discriminative features for defect detection. The experiment results show that the proposed *DSTEELNet* with three parallel stacks with different rates 1,2,3 achieved 97% *mAP* and outperformed state-of-the-art CNN architectures, such as *Yolov5*, *VGG16*, *VGG19*, *Resent50* and *MobileNet* with 8.8%, 6%, 7.2%, 4.2% and 3.2% higher *mAP*, respectively. In addition, we developed *DSTEELNet-ASSP* that improved the precision, recall and F1-score. As future research, we will explore methods to achieve more precise defect boundaries, such as performing defect segmentation based on deep learning techniques.

## Figures and Tables

**Table 1:** Image augmentation setting parameters applied to original NEU dataset.

Parameters	Value
Height Shift	0.08
Width Shift	0.08
Rotation Range	8
Fill mode	Nearest
Zoom Range	0.08
Shear Range	0.3

**Table 3:** Detection Results on GNEU dataset.

	*DSTEELNet*			*VGG16*			*VGG19*			*Resnet50*			*MobileNet*		
	Precision	Recall	F1	Precision	Recall	F1	Precision	Recall	F1	Precision	Recall	F1	Precision	Recall	F1
Crazing	1.00	1.00	1.00	1.00	1.00	1.00	0.95	1.00	0.97	0.99	1.00	0.99	0.98	1.00	0.99
Inclusion	0.97	0.86	0.91	1.00	0.51	0.68	0.94	0.54	0.69	1.00	0.66	0.79	1.00	0.82	0.82
Patches	1.00	1.00	1.00	0.89	1.00	0.94	1.00	0.98	0.99	1.00	0.99	0.99	1.00	0.99	0.99
Pitted_surface	0.87	0.97	0.92	0.66	0.97	0.79	0.67	0.89	0.76	0.74	0.98	0.84	0.73	0.98	0.84
Rolled-in_Scale	0.99	1.00	0.99	0.96	1.00	0.98	0.94	1.00	0.97	0.96	1.00	0.98	0.98	0.90	0.94
Scratches	1.00	1.00	1.00	1.00	0.87	0.93	1.00	0.99	0.99	1.00	0.98	0.99	0.96	1.00	0.98
***mAP***	**0.972**			0.912			0.90			0.93			0.94		

**Table 4:** Comparative results of single defect accuracy.

Defect	*DSTEELNet*	Yolov5	Yolov5-SE
Crazing	1.00	0.84	0.90
Inclusion	0.97	0.86	0.88
Patches	1.00	0.92	0.94
Pitted surface	0.87	0.89	0.99
Rolled-in scale	1.00	0.52	0.64
Scratches	0.99	0.98	1.00

**Table 5:** Weighted average results on Severstal dataset.

Model	Precision	Accuracy	F1-score
DSTEELNet	**0.96**	**0.96**	**0.96**
Vgg16	0.91	0.90	0.89
Vgg19	0.91	0.91	0.90
ResNet50	0.93	0.93	0.932
MobileNet	0.94	0.926	0.93

**Table 6:** Weighted average results for different DSTEELNet architectures with different dilated rates.

	DSTEELNet	Precision	Recall	F1-score
One Stack	*dilation 1,1,2,2,3*	0.93	0.93	0.93
	*dilation 1,2,3,4,5*	0.89	0.90	0.90
3-parallel stacks	*J = 1,2,3*	**0.96**	**0.958**	**0.957**
	*J = 2,3,4*	0.92	0.91	0.91
	*J = 1,2,5*	093	0.92	0.92
	*J = 1,2,3+ ASPP(4,10,16,22)*	*DSTEELNet-ASPP*		
		**0.98**	**0.98**	**0.98**

**Table 7:** Comparison of Computational time (ms) for traditional and deep learning techniques on GNEU dataset.

Traditional Techniques			Deep Learning Techniques					
HOG-SVM	LBP-SVM	GLCM- SVM	Vgg16	Vgg19	ResNet50	MobileNet	DSTEELNet	Yolov5
443.5	382.3	454.57	29	31	36	30	23	24

## Data Availability

Two publicly available datasets NEU and Serverstal to illustrate and evaluate the proposed architecture were used.

## Abbreviations

DL: Deep learning; CNN: Convolutional neural network; DSTEELNet: deep steel CNN-based architecture; *mAP*: mean average precision; *GNEU:* Generated NEU dataset, and *Severstal* dataset.
